# Timing of Blood Cultures in the Setting of Febrile Neutropenia: An Australian Institutional Experience

**DOI:** 10.4274/tjh.galenos.2020.2020.0302

**Published:** 2021-02-25

**Authors:** Samuel Wang

**Affiliations:** 1 Alexandra Hospital, National University Hospital System, Queenstown, Singapore

**Keywords:** Blood cultures, Febrile neutropenia, Neutropaenic sepsis, Microbiology, Haematology, Laboatory medicine

## Abstract

**Objective:**

Febrile neutropenia (FN) is a hematological emergency requiring urgent investigations to exclude infection and treatment with broad-spectrum antibiotics. Despite frequent blood cultures (BCs) being taking during episodes of FN, in the current literature BC positivity rates remain low in FN. This study aims to determine the BC positivity rate in FN hematology patients and determine the utility of collecting BCs beyond 24 h of commencing broad-spectrum antibiotics.

**Materials and Methods:**

BC results between 2014 and 2016 from all FN hematology patients were analyzed. Patient episodes of FN (PEFNs) were defined as a continuous period of FN where the interval between BC samples was a maximum of two days. In total from 2014 to 2016, 379 patients experienced 914 PEFNs and had 4267 BCs collected.

**Results:**

Overall BC positivity rates and BC-positive PEFN rates were 8.16% and 13.35%, respectively. Within the first 24 h, the positivity rate of the first BCs was 3.49%, while subsequent BC positivity within the first 24 h was 11.96%. BC positivity rates declined after 24 h to 2.18%.

**Conclusion:**

It is likely that BCs beyond 24 h of commencing broadspectrum antibiotics will rarely identify relevant microorganisms. Not collecting BCs after 24 h would likely reduce laboratory test costs, patient discomfort, and iatrogenic anemia.

## Abstract (tr)

**Amaç:**

Febril nötropeni (FN), enfeksiyonu ve geniş spektrumlu antibiyotiklerle tedaviyi dışlamak için ivedi araştırmalar gerektiren hematolojik bir acil durumdur. FN atakları sırasında sık kan kültürleri (KK) alınmasına rağmen, mevcut literatürde KK pozitiflik oranları FN’de düşük kalmaktadır. Bu çalışma, febril nötropenik hematoloji hastalarında KK pozitiflik oranını belirlemeyi ve geniş spektrumlu antibiyotiklere başladıktan 24 saat sonra KK almanın faydasını belirlemeyi amaçlamaktadır.

**Gereç ve Yöntemler:**

2014-2016 yılları arasında FN tanısı almış tüm hematoloji hastalarının KK sonuçları analiz edildi. Hasta FN atakları (HFNA), KK örnekleri arasındaki sürenin en fazla iki gün olduğu kesintisiz febril nötropenik dönem bir FN periyodu olarak tanımlandı. 2014’ten 2016’ya kadar toplamda 379 hastada 914 HFNA gözlendi ve 4267 KK örneği alındı.

**Bulgular:**

Genel KK pozitiflik oranları ve KK pozitif HFNA oranları sırasıyla %8,16 ve %13,35 idi. İlk 24 saat içinde, ilk KK’lerin pozitiflik oranı %3,49 iken, takip eden KK pozitifliği %11,96 bulundu. KK pozitiflik oranları 24 saat sonra %2,18’e geriledi.

**Sonuç:**

Geniş spektrumlu antibiyotikler başlamasını takiben ilk 24 saatten sonra alınan KK’lerin ilgili mikroorganizmaları tanımlaması nadiren muhtemeldir. Febril nötropenili hematoloji hastalarında geniş spektrumlu antibiyotik başlanmasını takiben ilk 24 saatten daha sonra KK’lerin alınmaması, laboratuvar test maliyetlerini, hastanın girişim anksiyetesini ve iyatrojenik anemiyi azaltacaktır.

## Introduction

Febrile neutropenia (FN) is defined as an oral temperature of >38.5 °C (or two consecutive readings of >38.0 °C for at least 2 h) with an absolute neutrophil count of <0.5x109/L (or expected to fall below 0.5x109/L) [1], and it is a medical emergency. FN in patients with hematological malignancies frequently complicates chemotherapy and is responsible for morbidity, increased healthcare resource utilization, and treatment delays compromising cancer treatment efficacy. Mortality from FN has diminished steadily but still remains significant at around 11% [1]. Patients with proven bacteremia have worse prognosis, with mortality rates of about 18% in cases of gram-negative and 5% in cases of gram-positive bacteremia [1].

Generally, patients presenting with FN are investigated for an infection source and are begun on empiric broad-spectrum antibiotic therapy as soon as practical. Investigations include blood cultures (BCs) from a central access catheter (CVC) if present as well as peripheral blood. The rates of positive BCs in FN patients are low, ranging from 0.2% to 15% [2,3]. The diagnostic yield for repeated BCs is also low [4]. Positive BC rates are influenced by several factors including prophylactic antibiotics, degree of bacteremia, and presence of CVCs [2]. Limited information has been published to provide guidance on the utilization of BCs in this clinical context.

Furthermore, false-positive results due to contaminant organisms may generate a spiral of additional laboratory testing, unnecessary antibiotic therapy, and prolonged hospital stays with increased costs and little clinical benefit to the patient [5,6,7]. Additionally, repeated collection of BCs generates patient discomfort and exacerbates iatrogenic anemia [8]. A previous study looking at antibiotic de-escalation strategies in cases of multidrug-resistant gram-negative bacilli (MDR-GNB) bloodstream infections 24 h after cultures were taken identified that the vast majority of BCs (92.1%) from onco-hematological patients with FN were positive within 24 h and no MDR-GNB was positive over 24 h [9]. This supports the need for reassessing empiric antibiotic treatment in neutropenic patients at 24 h in light of antibiotic stewardship de-escalation strategies [9].

Building upon previous studies and with a different focus, we aimed to better define a more reasonable utility of BCs in the setting of FN. Specifically, we aim to determine if taking BCs beyond the first 24 h of empiric antibiotic therapy has any clinical role.

## Materials and Methods

All BC results of adult hematology patients with FN admitted via the Emergency Department to the Prince of Wales Hospital in Randwick, Australia, in the calendar years of 2014 to 2016 were retrospectively obtained. Many patients had multiple and prolonged admissions for FN, with multiple BCs performed. Hence, each patient’s BC record was divided into patient episodes of FN. A single patient episode of FN (PEFN) was defined as a single continuous period of FN where there was a constellation of clinical symptoms such as fever, infectious focus, appearance of new symptoms and signs of hypotension, tachycardia, and temperature of >38.5 °C. Furthermore, such symptoms and signs were present prior to the taking of BCs. Some patients had multiple PEFNs during a single prolonged admission.

The 2014-2016 dataset included the date and time of BC collection, the site of collection (e.g., peripheral blood or CVC), and microbial analysis of all BCs taken for each patient. A standard protocol for managing patients presenting with FN has been in place for many years. All patients have BCs collected by peripheral venipuncture as well as all lumens of CVCs if present prior to commencement of empiric intravenous antibiotics (tazobactam with vancomycin if CVC in situ). When the time of commencement of antibiotics was not known, it was assumed to follow the first collection of BC. The analysis determined the overall rates of BC positivity or negativity for the first BC collected, all BCs collected within 24 h of the first BC collected, and all BCs collected 24 h after the first BC collected (assumed to be equivalent to 24 h of empiric antibiotic therapy). A table of microbial isolates was also constructed for the whole dataset. In addition, case records were reviewed in detail for patients where BCs were positive only after 24 h from the first BC collected.

## Results

In 2014-2016, a total of 379 patients experienced 930 PEFNs and had 4267 BCs collected, and the overall BC positivity rates and BC-positive PEFN rates were 8.16% and 13.12%, respectively (Table 1). Two-thirds of BCs (2801) were collected in the first 24 h, with the remainder (1466) being collected 24 h from the first BC. The first BC was positive in 3.49% of cases.

Based on the total number of PEFNs, a chi-square test was performed to compare the number of PEFNs, where the first BC taken within 24 h being positive (n=98) was compared to the number of PEFNs where any BC within 24 h was positive (n=115), and the chi-square statistic was 1.53 (p=0.21). Hence, there was no statistical difference in the number of PEFNs for the first BC taken within 24 h being positive versus the number of PEFNs for any BC being positive within 24 h. Additionally, for the 122 BC-positive PEFNs, the first BC identified 80.33% of cases, while BCs collected in the first 24 h identified 94.26% (Table 2). This implies that the first BC taken prior to the initiation of antibiotics would have the highest microbiological yield. However, it also suggests that any number of BCs can be taken within a 24 h period and this does not have to be limited to the first BC alone. This is useful for doctors who may want to consider taking BCs despite already starting empirical antibiotics, with the caveat of the 24 h period.

The BC positivity rates rapidly declined 24 h after commencement of broad-spectrum antibiotics. The positivity rates of all BCs within the first 24 h was 11.95% compared with 2.18% for all BCs taken more than 24 h after the first BC (chi-square statistic of 117.02, p<0.00001) (Table 3). This supports the idea that taking BCs after 24 h of empiric antibiotics is likely to have a low diagnostic yield. BCs within 24 h of admission and commencement of broad-spectrum antibiotics gave the highest yield of 56.80%, in contrast to only 12.15% of BCs being positive after 24 h (Table 4).

The BCs that were collected 24 h after the first BC were positive in only 17 episodes of FN. In 10 of these episodes of FN, BC positivity was also evident in the first 24 h and continued to be positive beyond the first 24 h. BC positivity was detected only after 24 h in only seven cases (Table 5). Based on a detailed clinical review of these cases, it is likely that contaminant microbes were found in 4 cases (6 of the 11 BCs). A summary of all microbial isolates is provided in Table 6. To better contextualize the blood culture findings, clinical details such as patient demographics, transplant status, underlying hematological disease, average duration of neutropenia, and average absolute neutrophil count on admission were collated (Table 7).

## Discussion

In this retrospective study, we analyzed 4267 BCs taken from 379 hematology patients admitted for FN between 2014 and 2016. This BC positivity rate of 8.16% is consistent with previous reports [3,4]. While small, this yield is clinically worthwhile and supports the use of BCs in patients with FN. Our results, however, indicate little clinical utility of BCs taken beyond 24 h from commencement of empiric antibiotics. Of 1466 BCs taken beyond 24 h of initial BC collection, 97.8% were negative. Most of the 2.2% of positive BCs taken beyond the first 24 h of empiric antibiotic therapy continued to be positive for bacteremia as already identified in the BCs taken within the first 24 h. In only 3 cases did the BC beyond the first 24 h identify potentially new pathogenic organisms. We conclude that further BCs should be taken within the first 24 h in cases of further spikes of temperature of ≥38.5 °C. However, BCs should not be routinely collected after 24 h of broad-spectrum antibiotic therapy unless there is clinical suspicion of undiagnosed infection. Our results largely reflect the limited published data found regarding BC timing in FN [10,11].

Our practice will hopefully save money in investigations and reduce the risk of unnecessary escalation of investigations/treatment due to microbial isolates from contaminated BCs [12]. Contaminated BCs may result in unnecessary treatment for the patient and extended hospital stay [12]. There is also greater financial burden from inappropriate and contaminated BCs, such as the costs of cultures, extended hospital stays, and pharmacy costs [12]. Contaminated BCs also have the unwanted effect of prolonging broad-spectrum antibiotic therapy, which can lead to greater antimicrobial resistance and also side effects such as *Clostridium difficile* infections [12]. Moreover, reduction in the amount/volume of BCs will help ameliorate patient discomfort and iatrogenic anemia in patients already suffering from multifactorial causes of anemia [8].

Based on our microbial isolates, the most common gram-positive organism was *Staphylococcus epidermidis*, followed by *Streptococcus salivarius*, *Staphylococcus hominis*, and *Staphylococcus aureus*. The most common gram-negative organism was *Escherichia coli*, followed by *Pseudomonas aeruginosa* and *Klebsiella pneumoniae*. In keeping with the current literature, our data suggest that there has been a shift from gram-negative bacteria towards gram-positive cocci in the majority of FN cases [13]. That being said, gram-negative bacteria such as *Escherichia coli*, *Pseudomonas aeruginosa*, and *Klebsiella *species still account for a large number of FN cases [13]. Reasons explaining such a shift could be increased oral mucositis associated with increasingly potent chemotherapeutic agents, such as cytosine arabinoside, or increasing use of indwelling intravascular catheters and fluoroquinolone prophylaxis resulting in decline in bacteremia secondary to gram-negative rods in FN, but not gram-positive organisms [13].

Unfortunately, data regarding antimicrobial resistance patterns were not able to be obtained from our bacterial isolates for this study. However, based on clinical experience and local antibiogram data, tazobactam is chosen as the first-line monotherapy for most patients with the addition of vancomycin only if the patient has a CVC in situ as a precaution against methicillin-resistant *Staphylococcus aureus* [14]. The rationale for this is combination therapy with an antipseudomonal beta-lactam and a second agent, typically an aminoglycoside, showing no clinical advantage over monotherapy with an antipseudomonal beta-lactam in meta-analyses of randomized controlled trials in sepsis [14]. Furthermore, nephrotoxicity with beta-lactam/aminoglycoside combination therapy usually outweighs any potential benefit [14].

## Conclusion

Our data demonstrate that BCs beyond 24 h of commencing broad-spectrum antibiotics rarely identify relevant microorganisms. Hence, BCs should not be routinely collected beyond 24 h of broad-spectrum antibiotic therapy unless there is strong clinical suspicion of undiagnosed infection.

There are a number of limitations to our study. The study is retrospective and the case records of all patients admitted with FN were not reviewed in greater detail due to limitations in the electronic health record; hence, the timing of the onset of symptoms and fever was not known. This could affect the accuracy of PEFNs. The use of prophylactic antibiotics prior to presentation, although likely to be small, was not known. The actual time of antibiotic administration in relation to the BC collection was also not known. However, the management of this patient group with a well-established long-standing protocol with collection of BCs followed by commencement of empiric antibiotics makes our conclusion reasonable.

Possible prospective future studies could eliminate these limitations and more accurately determine the number of BC sets required to most cost-effectively identify bacteremia in this clinical context.

## Figures and Tables

**Table 1:**
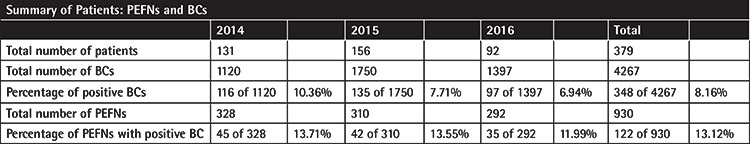
In the calendar years 2014-2016, 379 patients experienced 930 patient episodes of febrile neutropenia (PEFNs). A total of 4267 blood cultures (BCs) were collected, of which 348 (8.16%) were positive. Out of the total 930 PEFNs, 122 (13.35%) had positive BCs.

**Table 2:**
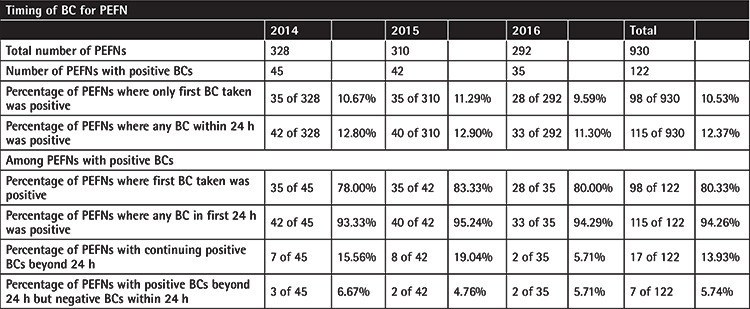
In 122 blood culture (BC)-positive patient episodes of febrile neutropenia (PEFNs), the first BC identified 80.33% of cases and BCs collected in the first 24 h identified 94.26%.

**Table 3:**
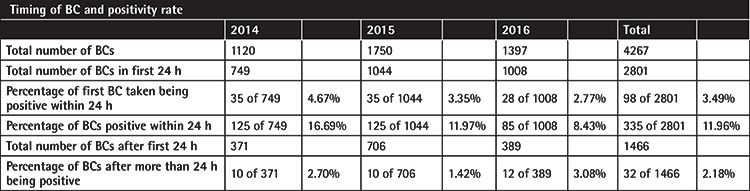
Positive blood culture (BC) rates were highest under 24 h at 11.96%. Beyond 24 h, the positive BC rate had a low value of 2.18%.

**Table 4:**
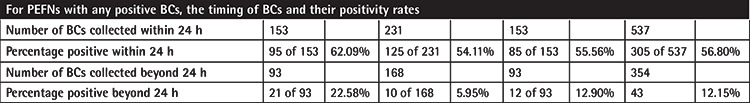
Analyzing patient episodes of febrile neutropenia (PEFNs) for the group with any positive blood cultures (BCs), the highest BC positivity rate was that for BCs taken within 24 h at 56.80%.

**Table 5:**
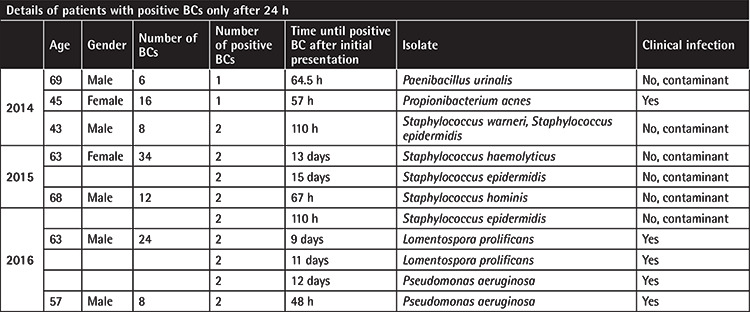
This table highlights specific patients who had positive blood cultures (BCs) after 24 h despite having negative BCs within the initial 24-h period. Half of the organisms isolated appeared to be contaminants and not clinically significant.

**Table 6:**
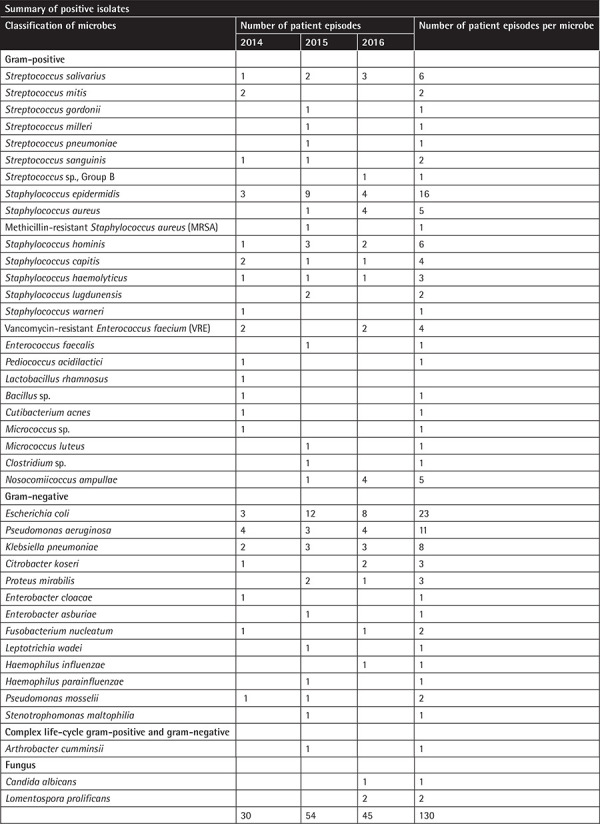
The isolated microorganisms from febrile neutropenia patients treated in our institution.

**Table 7:**
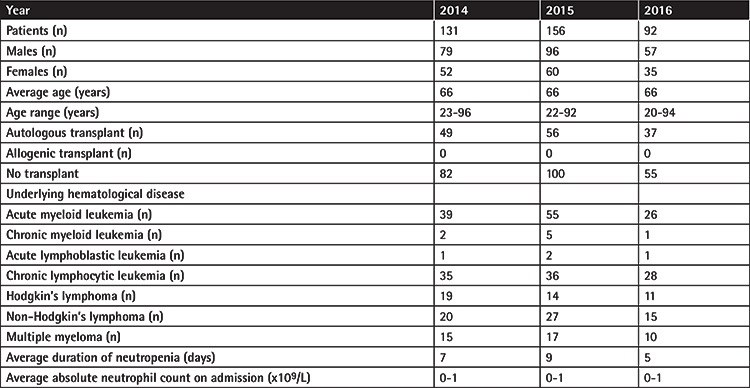
Relevant clinical data of the 379 adult patients admitted for febrile neutropenia in the calendar years of 2014-2016. (n) refers to the number of patients.
